# Immunogenicity and Efficacy of CpG-Adjuvanted In-Ovo Recombinant HVT-LT and FPV-LT Vaccines Against Infectious Laryngotracheitis Challenge in 14-Day-Old Broiler Chickens

**DOI:** 10.3390/v18101096

**Published:** 2026-10-03

**Authors:** Carissa Gaghan, Matthew Browning, Aakriti Tyagi, Pok Man Chan, Mohamed Faizal Abdul-Careem, Isabel M. Gimeno, Raveendra R. Kulkarni

**Affiliations:** 1 Department of Population Health and Pathobiology, College of Veterinary Medicine, North Carolina State University, 1060 William Moore Drive, Raleigh, NC 27606, USA; cegaghan@ncsu.edu (C.G.); mhbrowni@ncsu.edu (M.B.); atyagi7@ncsu.edu (A.T.); pchan3@ncsu.edu (P.M.C.); 2 Health Research Innovation Center 2C58, Faculty of Veterinary Medicine, University of Calgary, Calgary, AB T2N 4N1, Canada; mfabdulc@ucalgary.ca

**Keywords:** infectious laryngotracheitis, recombinant vaccine, protection, CpG oligonucleotide, adjuvant, chickens

## Abstract

Recombinant herpesvirus of turkey laryngotracheitis (rHVT-LT) and fowlpox virus (rFPV)-LT in-ovoin-ovo vaccines are used for ILT control in broiler chickens. Previously, we showed that in-ovoin-ovo CpG-oligodeoxynucleotide (CpG-ODN) adjuvantation of rHVT-LT can protect broilers against ILT at 28 days of age. Here, we evaluated the immunogenicity and protective efficacy of in-ovoin-ovo CpG-ODN-adjuvanted rHVT-LT and rFPV-LT in broilers when challenged at 14 days of age. Eighteen experimental groups incorporated CpG-ODN-only treatments or those receiving rHVT-LT or rFPV-LT with/without CpG-ODN or non-CpG control ODN in-ovoin-ovo, followed by ILTV challenge at 14 days of age. While none of the vaccinated groups provided significant clinical protection, rHVT-LT+CpG-ODN vaccination showed a numerical reduction (*p* = 0.0710) in disease severity following ILTV challenge compared with the unvaccinated control. CpG-ODN-only or CpG-ODN-adjuvanted vaccinations followed by challenge resulted in increased (*p* < 0.05) tracheal and/or splenic TLR21, IL-12, or IFNγ transcription, indicating TLR-mediated antiviral adjuvant effects. Furthermore, CpG-ODN+rHVT-LT/rFPV-LT groups receiving ILTV had increased (*p* < 0.05) splenic frequencies of macrophages, γδ T cells, B cells, and activated CD4^+^/CD8^+^ T cells. Collectively, our findings indicate that in-ovo CpG-ODN adjuvantation with recombinant-vectored ILT vaccines can induce measurable immune activation and a trend toward reduced ILT disease severity following early ILTV challenge in broiler chickens.

## 1. Introduction

Infectious laryngotracheitis (ILT) is an acute, highly contagious, and economically devastating upper respiratory tract disease of chickens caused by an alphaherpesvirus, belonging to the family Herpesviridae. ILT outbreaks, particularly in broiler flocks, cause significant economic losses due to increased mortality and poor bird performance [1,2]. Conventional live-attenuated vaccines, mainly chicken embryo-origin (CEO) vaccines, have been used successfully in providing protective immunity against virulent ILTV field strains [3,4]. However, the CEO vaccine carries an inherent risk of reverting to a more virulent phenotype following bird-to-bird passages in the field, and recombination between distinct attenuated ILTV vaccine strains seems to generate virulent field viruses, together contributing to the emergence of vaccine-derived virulent strains that complicate regional ILT epidemiology [2,5]. On the other hand, we and others have shown that the commercial recombinant vectored ILT vaccines (referred to hereafter as ‘rILT’), recombinant herpesvirus of turkey laryngotracheitis (rHVT-LT), and recombinant Fowlpox virus laryngotracheitis (rFPV-LT) vaccines that are devoid of regaining virulence ability can reduce the severity of clinical disease and improve bird performance [2,6,7]. However, the rILT vaccines are associated with reduced immunogenicity and delayed onset of immunity, resulting in inadequate protective immunity compared with the CEO vaccine [4,6,8,9]. This delay in protective immunity has practical implications for broiler flocks operating in high ILTV-pressure environments, where early exposure may occur before full immunity develops.

The Toll-like receptor (TLR) adjuvants have been shown to aid vaccines in inducing an earlier onset of immunity. CpG-oligodeoxynucleotides (CpG-ODN) are synthetic single-stranded DNA recognized by innate immune receptors [10]. In mammals, CpG-ODN are detected by TLR9, while avian TLR21 mediates immune recognition, driving transcriptional upregulation of interferon (IFN)γ, interleukin (IL)-12, IL-1β, and inducible nitric oxide synthase (iNOS), as well as the recruitment and activation of immune cells [11,12]. In-ovo CpG-ODN delivery can enhance innate and adaptive immune responses in chicks, reducing mortality from ILTV, influenza virus shedding, and Marek’s disease virus replication, while in vitro CpG-ODN treatment of chicken embryo fibroblasts has also been shown to reduce Marek’s disease virus replication, thus demonstrating its broader antiviral utility as a poultry vaccine adjuvant [13,14,15,16,17]. Recently, we reported two studies investigating the CpG-ODN effects in chickens when delivered in-ovo [18,19]. One-day-old broiler chicks that received CpG-ODN in-ovo had enhanced splenic and lung transcription of IFNγ, IL-1β, and iNOS genes, and augmented activation of T cells [18]. Subsequently, we investigated the efficacy of in-ovo CpG-ODN-adjuvanted rHVT-LT vaccination in broilers against ILTV challenge at 28 days of age and found that CpG-ODN adjuvantation of rHVT-LT can provide protective immunity [19]. The protection mechanisms included elevated tracheal and splenic expression of IL-12 and IFNγ genes and enhanced tissue recruitment of γδ T cells and activated CD4^+^ T cells. Considering the importance of early onset of ILT immunity in broilers, the present study evaluated the immunogenicity and protective efficacy of in-ovo CpG-ODN adjuvanted rHVT-LT and rFPV-LT in broilers when challenged at 14 days of age with a virulent ILTV field strain.

## 2. Materials and Methods

### 2.1. Animals and Experimental Design

**Figure 1:**
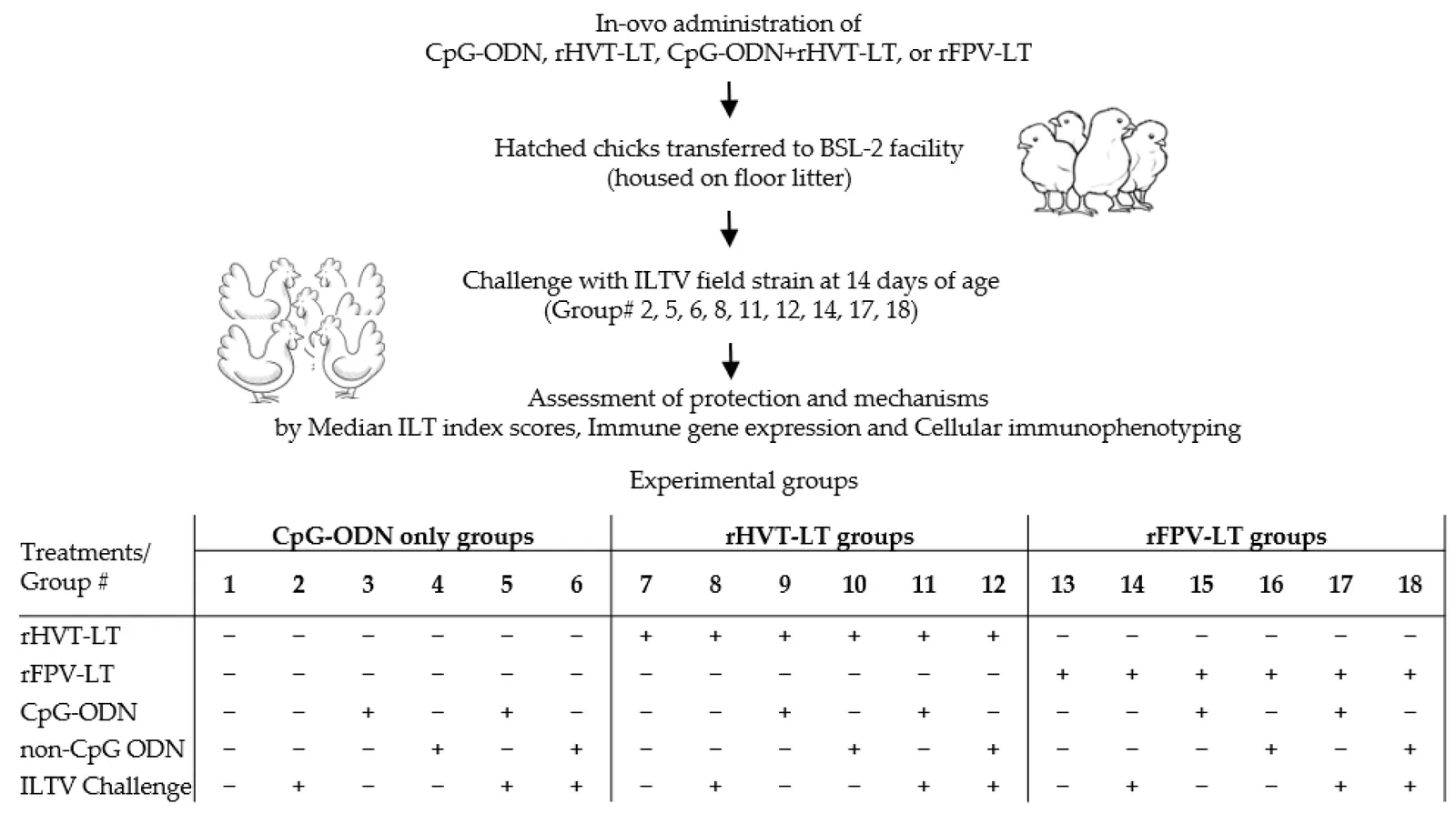
Experimental design. Schematic flow of experiments showing that embryonated eggs were in-ovo vaccinated at ED18 with either CpG-ODN alone, rHVT-LT ± CpG-ODN, or rFPV-LT ± CpG-ODN (10 µg per egg). Different treatment groups used in the study are tabulated at the bottom of the figure. Upon hatching, chicks were assigned individual bird numbers and housed in pens on floor litter in a BSL-2 facility. Groups 2, 5, 6, 8, 11, 12, 14, 17, and 18 were challenged with ILTV intra-tracheally at 14 days of age. Clinical signs and mortality were observed for a week post-challenge, followed by necropsy on day 21 of age to assess ILT index scores. Trachea and spleen were also collected at necropsy for immune gene expression using real-time PCR, and cellular immunophenotyping using flow cytometry.

All the animal experiment protocols used in this study were approved by the North Carolina State University Institutional Animal Care and Use Committee (IACUC, protocol 21-203-A). Fertile Ross 708 broiler breeder chicken eggs graciously provided by Aviagen Inc. (Huntsville, AL, USA) were incubated and used for this experiment. For each group, 35 embryonated eggs were randomly selected and assigned to 18 groups that received CpG-ODN alone, rHVT-LT ± CpG-ODN, rFPV-LT ± CpG-ODN, or a vaccine diluent at 18 days of embryonation (ED), via the intra-amniotic route (referred to hereafter as ‘in-ovo’). The experimental design and treatment groups are depicted in Figure 1. One-day-old chicks were individually identified by wing band and neck tag (National Band and Tag Company, Newport, KY, USA) before being placed on fresh, autoclaved wood shavings litter in BSL-2 facility animal rooms. The treatment groups (*n* = 20–23) were placed in different rooms to avoid any possible cross-contamination during the viral challenge period. At 14 days of age, treatment groups 2, 5, 6, 8, 11, 12, 14, 17, and 18 were challenged with ILTV via intratracheal administration, as described previously [6]. The birds were then monitored for mortality and clinical signs post-challenge and necropsied at 21 days of age to score ILT lesions and collect samples.

An ILT index scoring system was used to quantify the severity of disease in the broilers, as described previously [6]. Briefly, the ILT index score is a combination of the clinical signs (CS) (scored 0 to 6) and the gross lesions (GL) present in the trachea at termination (scored 0 to 4). To evaluate the CS, the birds were individually observed daily from days 15–21 to record any clinical signs of ILTV. The CS included gasping, coughing, sneezing, conjunctivitis, and expectoration. The CS calculated the number of days that a chicken was showing clinical signs. Tracheal GLs were classified in an objective rank from 0 to 4, being normal (0), presence of light mucus (1), congested, thick mucus or bloody mucus (2), presence of caseous exudate (3), and presence of a caseous plug (4). The ILT Index (ILTI) was calculated as the sum of the clinical signs (CS) score and the gross lesions (GL) score, with values ranging from 0 to 10 (Supplemental Figure S1). To minimize observer bias, lesion scoring was performed by an ACPV board-certified poultry veterinarian who was blinded to experimental group details.

### 2.2. Vaccines, Adjuvant, and Virus

Commercial rHVT-LT and rFPV-LT vaccines expressing the ILTV glycoprotein B (gB) and UL-32 proteins were obtained from Ceva Animal Health Inc. (Lenexa, KS, USA) and used for in-ovo administration at the recommended dose following the manufacturer’s instructions [2,8]. Synthetic CpG-ODN (ODN 2007, InvivoGen, San Diego, CA, USA), a class B CpG-ODN containing a full phosphorothioate backbone with one or more CpG dinucleotides, was used as the adjuvant. The lyophilized powder of CpG-ODN was dissolved in endotoxin-free water and stock solutions of 10 mg/mL were prepared and administered in-ovo at 10 µg per egg. The CpG-ODN was mixed with rHVT-LT or rFPV-LT vaccine in a volume of 100 µL/egg for in-ovo administration. The Illinois-N71851 virulent strain of ILTV was grown in the chorioallantoic membrane for one passage from the low-passage ATCC seed lot, titrated in chicken kidney cells, and used for the challenge [6]. Birds in challenge groups were administered ILTV (4000 PFU/bird) at 14 days of age via intratracheal inoculation.

### 2.3. Immune Analyses

To evaluate the effect of different treatments on immunological responses, tracheal and splenic immune gene expression and splenic cellular responses were quantified. Given the large number of experimental groups (*n* = 18), a subset of 6 birds per group was randomly selected for immunological analyses. This sample size was considered sufficient to capture representative immune gene expression and cellular response data across treatment groups while accommodating the overall scope of the study within available resources.

#### 2.3.1. Gene Expression

Tracheal and splenic samples were collected in RNAlater solution (Invitrogen, Carlsbad, CA, USA) from chickens in all the groups (*n* = 6), stored at −80° C and processed, as described previously [18,19]. The tissues were homogenized using a Bead Ruptor Elite Bead Mill Homogenizer (OMNI International, Kennesaw, GA, USA) with 1.4 mm Ceramic Beads (OMNI International, Kennesaw, GA, USA) and total RNA was extracted using TRIzol reagent (Invitrogen, Carlsbad, CA, USA) according to the manufacturer’s protocol before being treated with a DNA-free Kit (Invitrogen, Carlsbad, CA, USA). Subsequently, cDNA synthesis was performed with 1000 ng of purified RNA using a High-Capacity RNA-to-cDNA kit (Applied Biosystems, Waltham, MA, USA) according to the manufacturer’s recommended protocol. The resulting cDNA was diluted at a 1:10 ratio in nuclease-free water for real-time PCR analysis. Quantitative real-time PCR using SYBR Green was performed on diluted cDNA using a QuantStudio 6 Flex System and QuantStudio Real-Time PCR Software v1.6.1 (Applied Biosystems, Waltham, MA, USA) to quantify the cellular expression of TLR21, IL-1β, IL-12, IFNβ, IFNγ, and iNOS. Briefly, each reaction involved a pre-incubation period of 50 °C for 2 min followed by 95 °C for 2 min, followed by 35–45 cycles of 95 °C for 10 s, 58–64 °C for 5 s, depending on the primers’ binding suitability, and the elongation step was 72 °C for 10 s. Subsequent melt curve analysis was performed by heating to 95 °C for 15 s, cooling to 60 °C for 1 min, and heating to 95 °C for 15 s. Primers for the amplification of all genes were synthesized by Integrated DNA Technologies (Coralville, IA, USA), and the primer sequences are given in Table 1. Relative expression levels of all target genes were calculated relative to the reference gene β-actin [20].

#### 2.3.2. Flow Cytometry

Single-cell splenocyte preparations (*n* = 6 per group) were made following a protocol described previously [18]. The cells were stained in two panels of antibody staining due to the paucity of chicken antibody reagents available in multi-color formats. All the anti-chicken antibodies were purchased from Southern Biotech Inc., Birmingham, AL, USA, and were of mouse origin, and their respective clones are given in parentheses below. The first panel used antibodies against mannose receptor C1-like B (MRC1L-B) (monocyte/macrophage lineage, clone KUL01), γδTCR (TCR-1), IgM (M1), and Bu-1 (AV-20), and the second panel used anti-CD3 (CT-3), CD4 (CT-4), CD8 (CT-8 recognizing CD8α chain), and CD44 (AV6). In both panels, a cell viability dye, Live/Dead Near IR (Invitrogen, Carlsbad, CA, USA), was used to exclude dead cells. The chicken cell markers and their corresponding antibody fluorochrome formats are given in Table 2. The stained cells, along with the single-stain compensation and fluorescent minus one control, were washed and fixed with 4% paraformaldehyde before data acquisition using an LSR-II flow cytometer (BD Biosciences, Franklin Lakes, NJ, USA). The data analysis was carried out using the FlowJo software v10 (Tree Starr, Ashland, OR, USA). The gating strategy included the exclusion of doublet cells through forward and side scatter plotting using the area (A), height (H), and width (W), followed by gating on live cells. Briefly, an analysis of all the samples was performed on the basic ‘live cell’ gate and the cells expressing mannose receptor C1-like B (MRC1L-B) (monocyte/macrophage lineage, clone KUL01) were gated as macrophages, TCRγδ^+^ cells were gated as γδ T cells, and cells expressing both Bu-1 and IgM were gated as B cells (Supplemental Figure S2). To determine the frequencies of T cell subsets, CD4^+^, and CD8^+^, the live CD3^+^ population was used as the backbone gate. To evaluate T cell activation, the live CD3^+^ cell (T cell) population was used as the backbone gate, followed by gating on the CD4^+^ and CD8^+^ T cells expressing CD44^+^ activation molecules (Supplemental Figures S2 and S3).

### 2.4. Statistical Analysis

All the data were analyzed using GraphPad Prism v10 (GraphPad software, San Diego, CA, USA). Data distribution was assessed using the Shapiro–Wilk test. For normally distributed data, group differences were analyzed using one-way analysis of variance (ANOVA) followed by Tukey’s multiple-comparisons test. For non-normally distributed data, differences among groups were analyzed using the Kruskal–Wallis test followed by Dunn’s multiple-comparisons test. Specifically, ILT lesion scores were analyzed using the Kruskal–Wallis test followed by Dunn’s multiple-comparisons test, whereas immune gene expression and immunophenotyping data were analyzed according to their distribution using either one-way ANOVA with Tukey’s multiple-comparisons test or the Kruskal–Wallis test with Dunn’s multiple-comparisons test. A *p*-value < 0.05 was considered statistically significant.

## 3. Results

### 3.1. Protection Against ILTV Challenge

**Figure 2:**
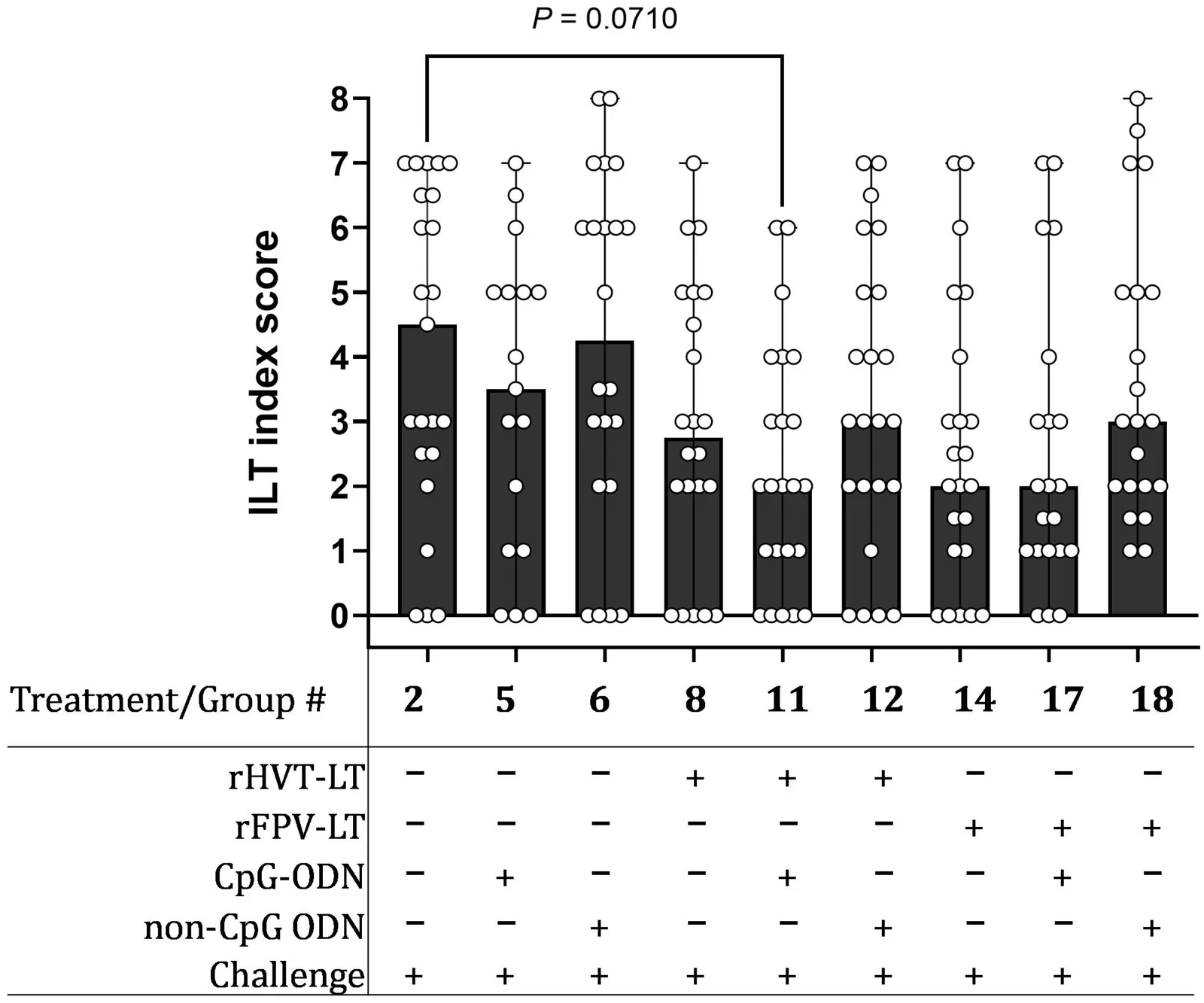
Assessment of vaccine-induced protection in chickens. Broiler chickens receiving in-ovo treatments with either CpG-ODN alone, rHVT-LT ± CpG-ODN, or rFPV-LT ± CpG-ODN were challenged intratracheally with wild-type ILTV at 14 days of age. ILT index score is a combination of the clinical signs (CS) (scored 0 to 6) and the gross lesions (GL) present in the trachea at termination (scored 0 to 4). The CS were recorded for each individual chicken for 6 days following ILTV challenge and the GL were scored at the time of death or at termination (7 days post-challenge). Each circle represents one bird. Data are presented as median with minimum–maximum range.

To evaluate the protective efficacy of the different treatment groups against the viral challenge, the clinical disease severity was quantified by ILT index scores (Figure 2). The unvaccinated chickens (Group 2) showed severe clinical disease based on the highest ILT indices amongst all the groups. Birds receiving rHVT-LT + CpG-ODN (Group 11) had a reduced (*p* = 0.0710) clinical score compared with the unvaccinated control (Group 2). A statistically non-significant numerical reduction in clinical disease severity was also observed in the rFPV-LT + ILTV (Group 14) and rFPV-LT + CpG-ODN + ILTV chickens (Group 17).

### 3.2. Immune Gene Expression

To evaluate the effect of different treatments on the tracheal and splenic immune responses, the expressions of TLR21, IL-1β, IL-12, IFNβ, IFNγ, and iNOS were quantified. For the ease of data interpretation and readability, the results described below are divided into three categories: (1) CpG-ODN only (Groups 1–6) to assess adjuvant-only effects with completely negative (Group 1) and ILTV-only (Group 2) controls, (2) rHVT-LT (Groups 7–12) to assess CpG-ODN adjuvant effects on rHVT-LT vaccination along with ILTV-only (Group 2) as the unvaccinated control, and (3) rFPV-LT (Groups 13–18) to assess CpG-ODN adjuvant effects on rFPV-LT vaccination along with ILTV-only (Group 2) as the unvaccinated control.

#### 3.2.1. CpG-ODN Only Effects

**Figure 3:**
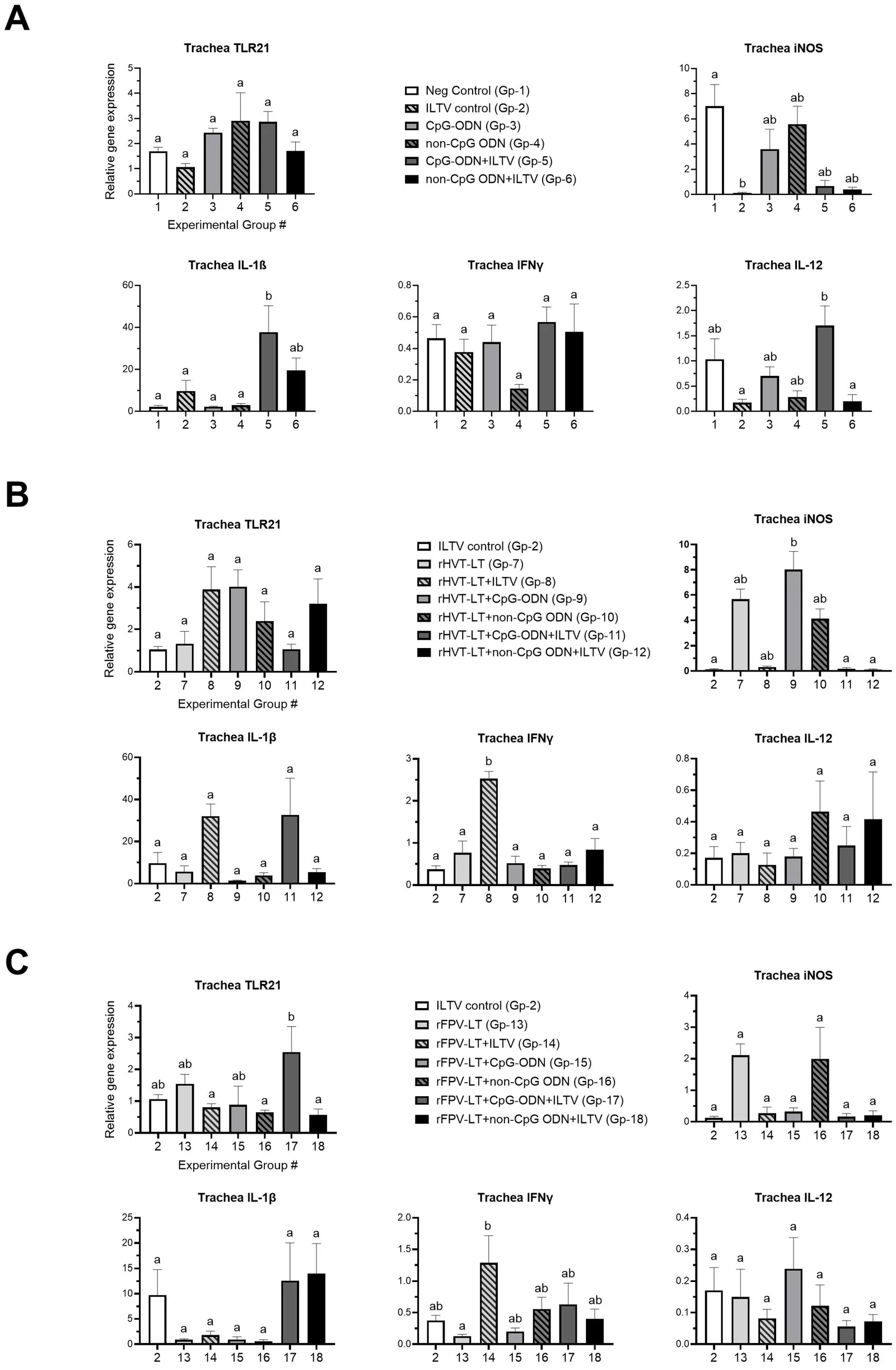
Effect of CpG-ODN alone and adjuvanted with rHVT-LT vaccine and rFPV-LT vaccine on the immune gene expressions in trachea of chickens challenged with ILTV. Broiler chickens receiving in-ovo treatments with CpG-ODN only (**A**), rHVT-LT ± CpG-ODN (**B**) and rFPV-LT ± CpG-ODN (**C**) were challenged intratracheally with wild-type ILTV at 14 days of age. Necropsy was performed on day 21 and tracheal samples were collected for RNA extraction and cDNA synthesis. Real-time PCR was performed to quantify immune gene expression along with the reference gene (β-actin) and the target gene expression levels relative to β-actin were calculated. Different letters above the standard-error-of-mean bars indicate significant differences (*p* < 0.05) between the groups.

**Figure 4:**
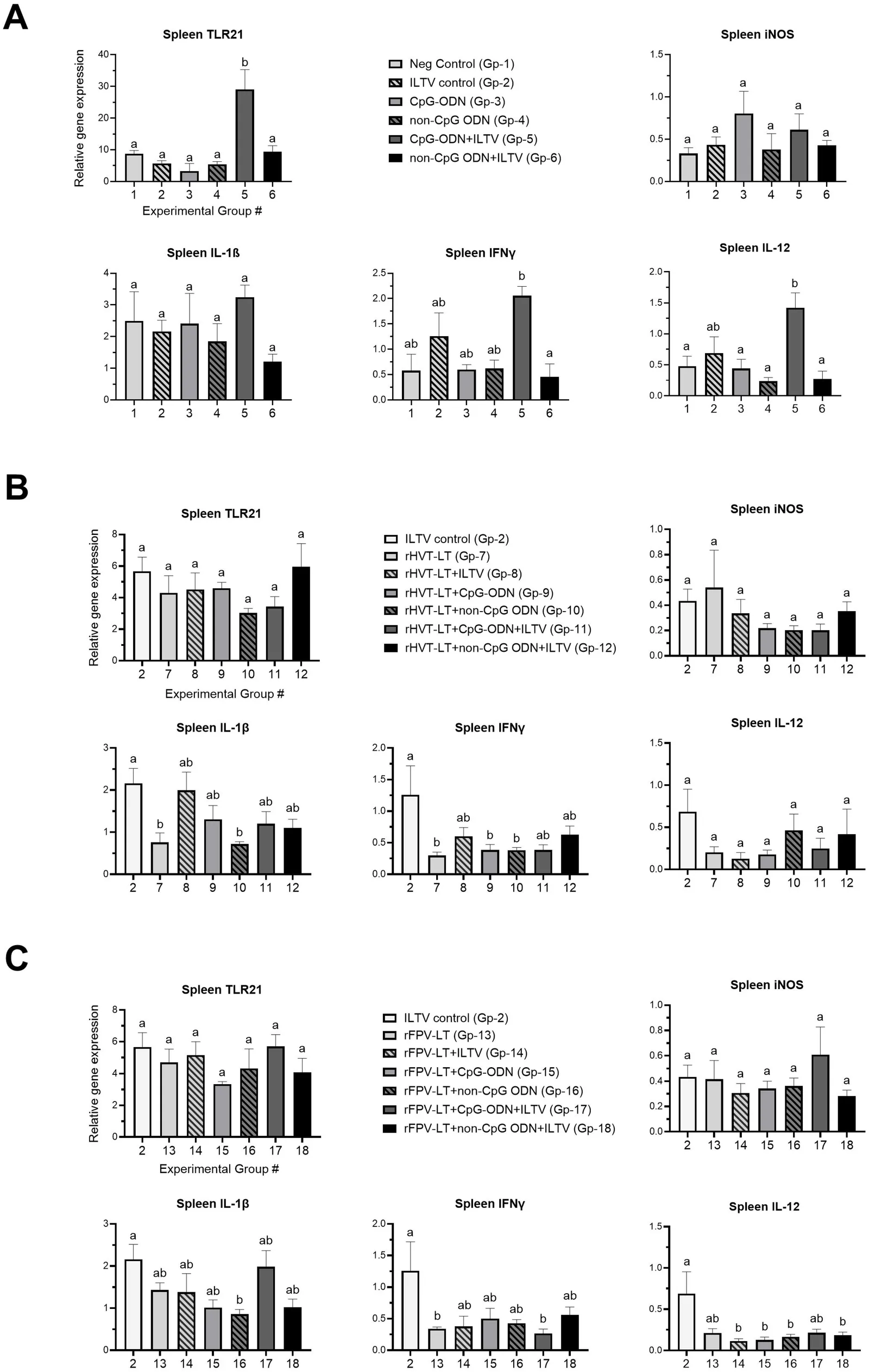
Effect of CpG-ODN alone and its adjuvancy with rHVT-LT vaccine and rFPV-LT vaccine on immune gene expression in the spleen of chickens challenged with ILTV. Broiler chickens receiving in-ovo treatments with CpG-ODN only (**A**), rHVT-LT ± CpG-ODN (**B**) and rFPV-LT ± CpG-ODN (**C**) were challenged intratracheally with wild-type ILTV at 14 days of age. Necropsy was performed on day 21 and splenic samples were collected for RNA extraction and cDNA synthesis. Real-time PCR was performed to quantify immune gene expression, along with the reference gene (β-actin), and the target gene expression levels relative to β-actin were calculated. Different letters above the standard-error-of-mean bars indicate significant differences (*p* < 0.05) between the groups.

As shown in Figure 3A, the tracheal expression of the iNOS gene in the ILTV control (Group 2) was reduced (*p* < 0.05) compared with the negative control (Group 1). The IL-1β expression was found to be elevated (*p* < 0.05) in the trachea of CpG-ODN + ILTV (Group 5) birds compared with all other experimental groups, except for the non-CpG-ODN + ILTV (Group 6), while IL-12 transcription was also found to be increased (*p* < 0.05) in Group 5 compared with ILTV control (Group 2) and non-CpG-ODN + ILTV (Group 6) birds. No significant changes were observed in the tracheal expression of TLR21 or IFNγ genes between the groups. In the spleen (Figure 4A), TLR21 transcription in the CpG-ODN + ILTV (Group 5) was higher (*p* < 0.05) compared with the rest of the groups, while Group 5 also had elevated (*p* < 0.05) gene expression of IFNγ compared with non-CpG-ODN + ILTV (Group 6), and IL-12 compared with the rest of the groups except ILTV-only control (Group 2). No significant changes in the splenic expression of iNOS or IL-1β genes were observed between the treatment groups. Furthermore, no significant changes in the tracheal or splenic expression of IFNβ gene were observed between the treatment groups. 

#### 3.2.2. CpG-ODN Adjuvant Effects on rHVT-LT Vaccination

As shown in Figure 3B, the tracheal expression of iNOS was higher (*p* < 0.05) in the rHVT-LT + CpG-ODN (Group 9) group compared with the ILTV-only control (Group 2), rHVT-LT + CpG-ODN + ILTV (Group 11), or rHVT-LT + non-CpG-ODN + ILTV (Group 12). The tracheal IFNγ was elevated (*p* < 0.05) in the rHVT-LT + ILTV group (Group 8) compared with all other groups. No significant changes were observed in the tracheal expression of TLR21, IL-1β, or IL-12 genes between the groups. In the spleen (Figure 4B), IL-1β transcription in the rHVT-LT only (Group 7) and rHVT-LT + non-CpG-ODN (Group 10) was reduced (*p* < 0.05) compared with the ILTV-only control (Group 2), while the rHVT-LT only (Group 7), rHVT-LT + CpG-ODN (Group 9) and rHVT-LT + non-CpG-ODN (Group 10) had reduced (*p* < 0.05) gene expression of IFN-γ compared with ILTV-only control (Group 2). No significant changes in the splenic expression of TLR21, iNOS, or IL-12 genes were observed between the treatment groups.

#### 3.2.3. CpG-ODN Adjuvant Effects on rFPV-LT Vaccination

As shown in Figure 3C, TLR21 transcription was significantly (*p* < 0.05) elevated in the rFPV-LT + CpG-ODN + ILTV group (Group 17) compared with the rFPV-LT + ILTV birds (Group 14), rFPV-LT + non-CpG-ODN (Group 16) and rFPV-LT + non-CpG-ODN + ILTV groups (Group 18). The expression of tracheal IFNγ was significantly higher (*p* < 0.05) in rFPV-LT + ILTV birds (Group 14) compared with unchallenged rFPV-LT-only birds (Group 13). No significant changes were observed in the tracheal expression of iNOS, IL-1β and IL-12 (*p* > 0.05). In the spleen (Figure 4C), the IL-1β expression was found to be reduced (*p* < 0.05) in the rFPV-LT + non-CpG-ODN (Group 16) birds compared with the ILTV-only control (Group 2). The transcription levels of splenic IFN-γ were lower (*p* < 0.05) in rFPV-LT-only (Group 13) and rFPV-LT + CpG-ODN + ILTV groups (Group 17) compared with the unvaccinated ILTV-only control (Group 2). Compared with the ILTV-only control group (Group 2), splenic IL-12 transcription was reduced (*p* < 0.05) in all rFPV-LT-vaccinated groups except the rFPV-LT-only (Group 13) and rFPV-LT + CpG-ODN + ILTV groups (Group 17). No significant changes in the splenic expression of TLR21 or iNOS genes were observed between the treatment groups.

### 3.3. Cellular Responses

Immunophenotyping analysis was carried out to quantify the splenic frequencies of macrophages (KUL-01^+^), B cells (Bu-1^+^IgM^+^), and γδ T cells (γδ TCR^+^) and CD4^+^, CD8^+^, and activated (CD44^+^) CD4^+^/CD8^+^ T cells. For the ease of data interpretation and readability, the results described below are divided into three categories: (1) CpG-ODN only (Groups 1–6) to assess adjuvant-only effects with completely negative (Group 1) and ILTV-only (Group 2) controls, (2) rHVT-LT (Groups 7–12) to assess CpG-ODN adjuvant effects on rHVT-LT vaccination along with ILTV-only (Group 2) as the unvaccinated control, and (3) rFPV-LT (Groups 13–18) to assess CpG-ODN adjuvant effects on rFPV-LT vaccination along with ILTV-only (Group 2) as the unvaccinated control.

#### 3.3.1. CpG-ODN Only Effects

**Figure 5:**
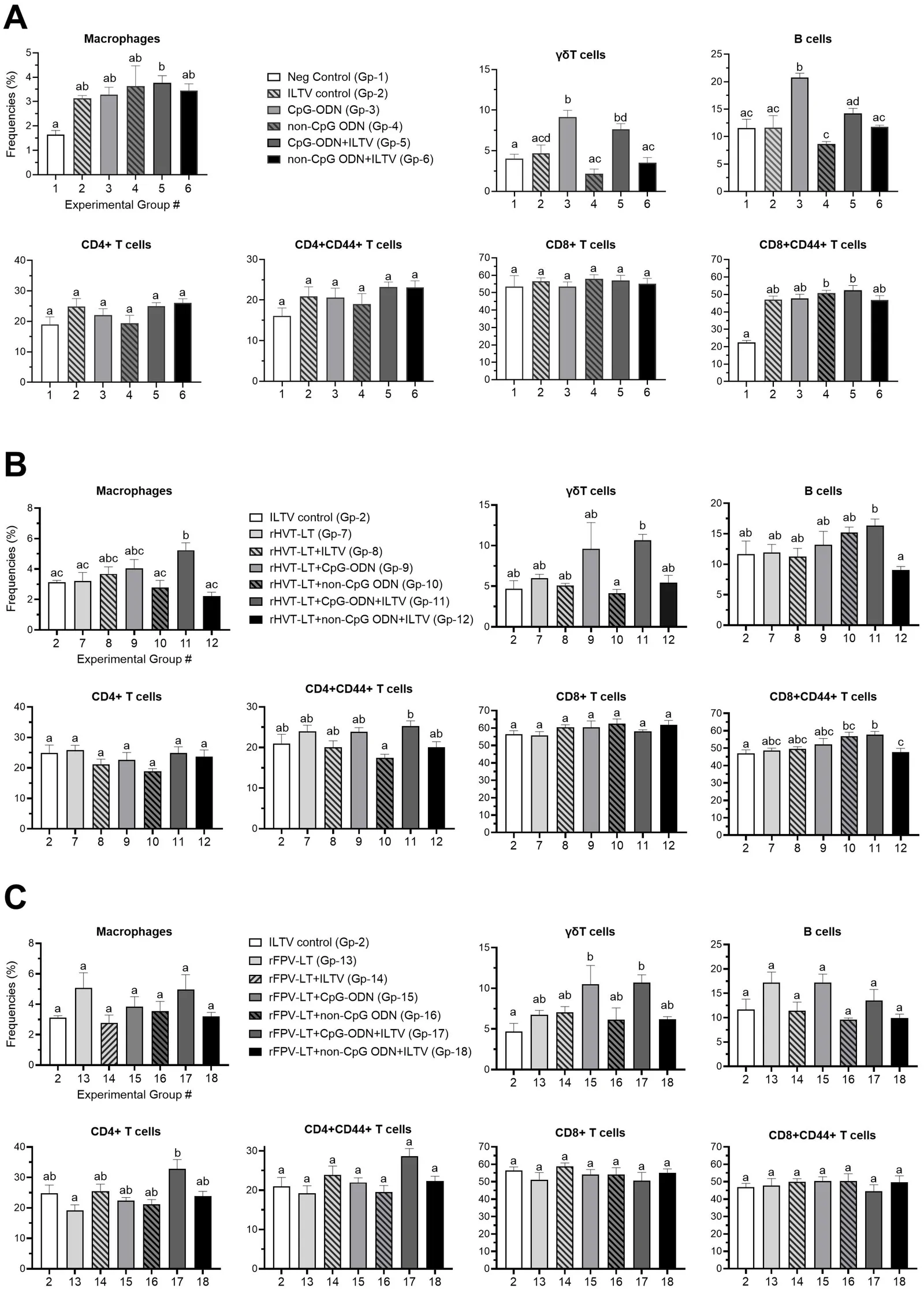
Effect of CpG-ODN alone and adjuvanted rHVT-LT vaccine and rFPV-LT vaccine on the splenic cellular responses in chickens challenged with ILTV. Broiler chickens receiving in-ovo treatments with CpG-ODN only (**A**), rHVT-LT ± CpG-ODN (**B**) and rFPV-LT ± CpG-ODN (**C**) were challenged intratracheally with wild-type ILTV at 14 days of age. Necropsy was performed on day 21 and splenic samples were collected for preparing single-cell suspensions. Cells were stained with antibodies against chicken KUL-01 (macrophages), Bu-1 and IgM (B cell markers), TCRγδ (γδ T cell), CD3, CD4, CD8, and CD44 (activation marker) molecules for flow cytometry-assisted immunophenotyping analysis. The bar graphs show the average cellular frequency of each of the cell populations. Different letters above the standard error of the mean bars indicate significant differences (*p* < 0.05) between the groups.

As shown in Figure 5A, CpG-ODN + ILTV (Group 5) birds had a higher (*p* < 0.05) macrophage frequency compared with the negative control (Group 1). CpG-ODN only (Group 3) treatment resulted in an increased (*p* < 0.05) γδ T cell frequency compared with the rest of the groups except CpG-ODN + ILTV (Group 5), while CpG-ODN + ILTV (Group 5) had an increased (*p* < 0.05) frequency of these cells compared with the rest of the groups except CpG-ODN only (Group 3) and ILTV control (Group 2). The CpG-ODN only (Group 3) treatment also resulted in a higher (*p* < 0.05) frequency of B cells compared with the rest of the groups, while CpG-ODN only (Group 3) and CpG-ODN + ILTV (Group 5) birds had an increased frequency of these cells compared with non-CpG-ODN only treatment (Group 4). The frequency of CD8^+^CD44^+^ cells was increased (*p* < 0.05) in non-CpG-ODN-only treatment (Group 4) and CpG-ODN + ILTV (Group 5) compared with the negative control (Group 1). No significant changes were observed in the frequency of CD4^+^ or CD8^+^ T cells between the treatment groups.

#### 3.3.2. CpG-ODN Adjuvant Effects on rHVT-LT Vaccination

As shown in Figure 5B, rHVT-LT + CpG-ODN + ILTV group (Group 11) birds had a higher (*p* < 0.05) macrophage frequency compared with the rest of the groups, except rHVT-LT + ILTV (Group 8) and rHVT-LT + CpG-ODN (Group 9). The rHVT-LT + CpG-ODN + ILTV group (Group 11) treatment resulted in an increased (*p* < 0.05) frequency of γδ T cells compared with the rHVT-LT + non-CpG-ODN group (Group 10). The rHVT-LT + CpG-ODN + ILTV (Group 11) treatment resulted in a higher (*p* < 0.05) frequency of B cells compared with the rHVT-LT + non-CpG-ODN + ILTV (Group 12). There was an increased frequency of CD4^+^ CD44^+^ T cells in rHVT-LT + CpG-ODN + ILTV (Group 11) as compared with rHVT-LT + non-CpG-ODN (Group 10). The frequency of CD8^+^CD44^+^ cells was increased (*p* < 0.05) in rHVT-LT + CpG-ODN + ILTV (Group 11) as compared with the ILTV control (Group 2) and the rHVT-LT + non-CpG-ODN + ILTV group (Group 12). No significant changes were observed in the frequency of CD4^+^ or CD8^+^ T cells between the treatment groups.

#### 3.3.3. CpG-ODN Adjuvant Effects on rFPV-LT Vaccination

As shown in Figure 5C, the rFPV-LT + CpG-ODN group (Group 15) and the rFPV-LT + CpG-ODN + ILTV group (Group 17) treatments resulted in an increase in γδ T cell frequency (*p* < 0.05) compared with the ILTV control group (Group 2). There was an increased frequency of CD4^+^ T cells in the rFPV-LT + CpG-ODN + ILTV group (Group 17) as compared with the rFPV-LT-only group (Group 13). No significant changes were observed in the frequency of macrophages, B cells, CD4^+^CD44^+^ or CD8^+^CD44^+^ T cells between the treatment groups.

## 4. Discussion

The broiler chicken industry forms the backbone of supplying protein nutrition to the growing global population. Therefore, broiler health is of paramount importance in sustainable poultry agriculture. However, ILT, one of the most important respiratory diseases affecting broilers, causes significant economic losses and demands newer, safer, and efficacious vaccines [1,3]. Although commercial in-ovo rHVT-LT or rFPV-LT vaccines can control ILT to a certain degree, the major problem with these vaccines has been poor immunogenicity and protective ability, and delayed onset of immunity. Our previous work has shown that adjuvanting in-ovo rHVT-LT vaccine with CpG-ODN adjuvant can boost immunogenicity and protect broilers against ILTV challenge at 28 days of age [19]. The present study evaluated the immunogenicity and protective efficacy of CpG-ODN-adjuvanted rHVT-LT and rFPV-LT in-ovo vaccines in broilers challenged at 14 days of age with ILTV.

Although none of the vaccinated groups provided significant clinical protection against ILTV challenge in broilers at 14 days of age, the primary finding of this study was that vaccination with rHVT-LT + CpG-ODN induced measurable immune activation and was associated with a numerical reduction (*p* = 0.0710) in ILT disease severity following virus challenge. This observation suggests that CpG-ODN may contribute to an earlier development of vaccine-induced immune responses. This possibility has practical relevance for commercial poultry production, where early development of effective immunity following in-ovo vaccination is desirable for controlling ILT during the early life of broiler chickens. The present observations are consistent with our previous findings demonstrating the immunopotentiating effects of CpG-ODN in broilers and showing that CpG-ODN-adjuvanted rHVT-LT administered in-ovo provided significant protection against ILTV challenge at 28 days of age [19]. Thus, the findings from the present and previous studies collectively suggest that CpG-ODN adjuvantation can enhance vaccine-induced immune responses and may contribute to earlier development of protection following in-ovo vaccination. However, the present study does not establish the precise onset of protective immunity, and the numerical reduction in disease severity observed at 14 days of age should be interpreted as an indication of a potentially developing protective response rather than as evidence of established clinical protection; further studies are warranted to establish the precise onset of protective immunity with and without CpG. Although statistically not significant, rFPV-LT + CpG-ODN vaccination also yielded an appreciable reduction in clinical disease severity, supporting the potential use of TLR ligands as rILT vaccine adjuvants [21,22].

In chickens, CpG-ODN is recognized by TLR21, leading to activation of antigen-presenting cells and downstream production of IL-12, which, in turn, drives IFNγ secretion by T cells toward promoting antiviral immunity [23,24]. In the present study, CpG-ODN, when used without rILT vaccines, yielded two key findings. (1) Enhanced early innate sensing and Th1 polarization following ILTV challenge, as determined by increased transcription of splenic TLR21, IL-12, and IFNγ, and tracheal IL-1β and IL-12 genes. Increased IL-1β transcription in the trachea further indicated an augmented local inflammasome-associated response that may aid in recruiting immune cells to the site of infection [13,22,25]. Furthermore, CpG-ODN alone increased γδ T cells and B cells, and upon ILTV challenge, this effect expanded to include macrophages and activated (CD44^+^) CD8^+^ T cells, indicating broad activation of innate and early adaptive immune components. Our previous work also noted enhanced innate and adaptive immune effects in one-day-old broiler chicks that were given CpG-ODN in-ovo [18]. (2) Birds that were untreated but ILTV-challenged had reduced expression of tracheal iNOS, IL-1β, and IL-12, suggesting attenuation of selected innate immune responses following ILTV infection. Because effective innate responses involving iNOS and pro-inflammatory cytokines can contribute to antiviral defense against ILTV, this reduction may represent a host response that is permissive to viral replication and persistence. Such an interpretation is consistent with the broader capacity of herpesviruses to modulate host immune responses and establish persistence [26,27]. Collectively, these observations suggest that CpG-ODN can effectively prime mucosal innate activation and Th1-mediated responses upon ILTV challenge, potentially contributing to improved antiviral immunity.

The CpG-ODN has been successfully employed as a vaccine adjuvant in chickens, demonstrating its ability to enhance both innate and adaptive transcriptional and cellular immune responses against viral pathogens, including ILT [13,16,17,18,19]. The present study evaluating the CpG-ODN adjuvant effect in broilers when administered in-ovo with rHVT-LT followed by ILTV challenge at 14 days of age yielded three key findings. (1) Birds receiving rHVT-LT + CpG-ODN had increased tracheal iNOS gene expression compared with ILTV-only, suggesting possible adjuvant-mediated macrophage responses at respiratory mucosal surfaces [28]. (2) Reduced transcription of splenic IFNγ and IL-1β genes in rHVT-LT + CpG-ODN vaccinated groups that did not receive ILTV challenge suggested a diminished systemic inflammatory response in favor of the host to control excessive pathology [19,29]. (3) Birds receiving rHVT-LT + CpG-ODN followed by ILTV challenge had increased macrophage, γδ T cell, and B cell populations, along with increased frequencies of activated (CD44^+^) CD4^+^ and CD8^+^ T cells. This observation suggests enhanced innate and adaptive immune cell responses, which may have contributed to a numerical trend toward reduced ILT disease severity following an early ILTV challenge in 14-day-old broiler chickens, although the observed reduction in clinical disease was not statistically significant (*p* = 0.071). It is noteworthy that CD44 is an adhesion receptor involved in the activated T cell migration between lymphoid tissues and the site of infection [30]. The key role of activated CD4^+^ and CD8^+^ T cells in defense against avian viruses, including ILTV, has been previously reported [30,31,32]. Additionally, our previous work found that rHVT-LT + CpG-ODN vaccinated broiler birds showing clinical protection against ILTV challenge at 28 days of age had increased splenic CD4^+^CD44^+^ T cells and γδTCR^+^ T cells [19]. Together, these observations indicate that CpG-ODN, when administered in-ovo as an rHVT-LT vaccine adjuvant, effectively modulates spatial immune gene expression and enhances cellular responses, resulting in an early onset of protective immunity against ILTV in broiler birds.

The in-ovo addition of CpG-ODN to the rFPV-LT vaccination seems to enhance mucosal innate sensing while modulating downstream systemic Th1 response following ILTV challenge. Increased tracheal TLR21 gene expression in the CpG-ODN + rFPV-LT + ILTV challenge group supported enhanced recognition of CpG motifs at the respiratory mucosa, consistent with the established role of TLR21 as the functional homolog of mammalian TLR9 in chickens [23]. The reduced splenic IFNγ transcription in rFPV-LT + CpG-ODN + ILTV-challenged birds, compared with ILTV-only controls, suggested a possible modulation of the systemic Th1-associated response following vaccination and challenge. This finding may reflect a more regulated systemic cytokine response in vaccinated birds while maintaining protective local immune responses [21,22,32]. TLR ligands, including CpG-ODN signaling via TLR21 in chickens, can modulate antiviral immunity through activation of innate immune pathways through TLR21, resulting in activation of immune-related genes and cytokine responses [22,25,33]. Furthermore, similar to rHVT-LT adjuvantation, chickens receiving rFPV-LT + CpG-ODN had increased γδ T cells and further elevated γδ T and CD4^+^ T cells following ILTV challenge compared with ILTV-only, rFPV-LT only, or non-CpG-ODN control groups. These findings are consistent with previous reports demonstrating that CpG-ODN enhances cellular immunity in chickens, and its ability to immunostimulate or immunomodulate host responses supports its function as an effective vaccine adjuvant [13,25,34,35]. Although rFPV-LT + CpG-ODN vaccination induced immune modulation and enhanced cellular responses, these changes provided limited clinical benefit following ILTV challenge at 14 days of age. This discrepancy suggests that immune activation alone may not translate into protection. Several factors may have contributed, including differences in vector replication, antigen expression kinetics, tissue tropism, and FPV immunogenicity in-ovo delivery [2,3]. The presence of maternal antibodies against FPV may have limited vaccine virus replication and antigen expression, reducing vaccine-induced immunity. In addition, the short interval between vaccination and challenge may have been insufficient for the development of a mature protective response [9]. Thus, although CpG-ODN may have enhanced immune activation, the magnitude, timing, localization, and quality of these responses may not have been sufficient for significant protection.

It is also noteworthy that the present work has certain limitations. (1) Viral load was not directly quantified, which could have provided additional insight into vaccine responses and virus replication dynamics. (2) Humoral responses were not assessed and future antibody-based assays would provide a more comprehensive understanding of treatment-specific immune responses. (3) Immunological analyses were performed on 6 birds per group to accommodate the large number of experimental groups (*n* = 18) within the scope and resources of this study. While sufficient to capture overall trends in immune gene expression, larger sample sizes in future studies would allow for more robust statistical power and finer resolution of group-level differences. (4) Following ILTV challenge, infection markedly upregulated immune gene transcription in both CpG-ODN–treated and untreated groups, likely masking CpG-ODN–specific effects against an already activated immune background. Consequently, differences observed in unchallenged birds became less pronounced post-challenge, with few statistically significant differences between treatment groups. (5) The relatively high cost of CpG-ODN should also be considered as a potential limitation for its large-scale commercial application in poultry. Nevertheless, the numerical reduction in disease severity observed in the CpG-ODN–adjuvanted rHVT-LT group, together with the associated immune responses, suggests a potential adjuvant effect of CpG-ODN and warrants further optimization of its dose, formulation, and route of administration.

## 5. Conclusions

In-ovo administration of CpG-ODN in combination with rHVT-LT enhanced vaccine-associated immune responses in broiler chickens and was associated with a numerical reduction in disease severity following ILTV challenge at 14 days of age, although this difference did not reach statistical significance. These findings suggest that CpG-ODN adjuvantation may facilitate an early development of vaccine-induced immune responses and may contribute to the subsequent development of ILT immunity. However, the present data do not establish protective immunity at 14 days of age. Together with the significant protection observed at 28 days of age in a previous study, the present findings support further evaluation of CpG-ODN as an adjuvant for improving the kinetics and magnitude of immune responses elicited by in-ovo rHVT-LT vaccination.

## Figures and Tables

**Table 1:** Primer Sequences used in real-time PCR.

Target Gene	Primer Sequence (5′-3′)	Annealing Temp (°C)	GenBank Accession
TLR21	F-CCTGCGCAAGTGTCCGCTCA R-GCCCCAGGTCCAGGAAGCAG	60	NM_001030558.1
IL-1β	F-GTGAGGCTCAACATTGCGCTGTA R-GTCCAGGCGGTAGAAGATGAAG	64	AJ009800
IL-12	F-CCAAGACCTGGAGCACACCGAAG R-CGATCCCTGGCCTGCACAGAGA	64	AY262752.1
IFNβ	F-GCCTCCAGCTCCTTCAGAATACG R- CTGGATCTGGTTGAGGAGGCTGT	64	X92479
IFNγ	F-ACACTGACAAGTCAAAGCCGCACA R-AGTCGTTCATCGGGAGCTTGGC	60	X99774
iNOS	F-GGCAGCAGCGTCTCTATGACTTG R-GACTTTAGGCTGCCCAGGTTG	64	NM 204961
Β-actin	F-CAACACAGTGCTGTCTGGTGGTA R-ATCGTACTCCTGCTTGCTGATCC	58	X00182

**Table 2:** Staining panels used in immunophenotyping.

	Marker	Conjugate
Panel 1	KUL01	PE
	**γ**δTCR	FITC
	IgM	APC
	Bu-1	Pacific Blue
	Live/Dead viable dye	APC-Cy7
Panel 2	CD3	Pacific Blue
	CD4	FITC
	CD8	PE
	CD44	APC
	Live/Dead viable dye	APC-Cy7

## Data Availability

Research data are available upon request from R.R.K.

## Supplementary Materials

The following supporting information can be downloaded at: https://www.mdpi.com/article/10.3390/v18101096/s1, **Figure S1:** Clinical data scoring. An ILT index scoring system was used to quantify the severity of the disease in the vaccinated broilers following ILTV challenge. The the ILT index score was calculated as the sum of the clinical signs (CS) (scored 0 to 6) and the gross lesions (GL) present in the trachea at termination (scored 0 to 4). **Figure S2:** Gating strategy for immunophenotyping γδ T cells, macrophages and B cells. The gating strategy included the exclusion of doublet cells through forward and side scatter plotting use the area (A), height (H), and width (W), followed by gating on live cells. The analysis of samples was performed on the ‘live cell’ gate, and the cells expressing KUL01 were designated as macrophages, and TCRγδ+ cells designated gated as gd (γδ) T cells, and while cells expressing both Bu-1 and IgM were designated as B cells. **Figure S3:** Gating strategy for analyzing CD4+ and CD8+ T cells. The gating strategy included the exclusion of doublet cells through forward and side scatter plotting use the area (A), height (H), and width (W), followed by gating on live cells. The sample analysis was performed on the ‘live cell’ gate to obtain CD4+ and CD8+ cell populations. Furthermore, cells expressing CD4 and CD44 were designated as activated CD4+ T cells, while those expressing CD8 and CD44 were designated as activated CD8 T cells.

## References

[1] Dufour-Zavala L. (2008). Epizootiology of infectious laryngotracheitis and presentation of an industry control program. Avian Dis..

[2] Garcia M., Zavala G. (2019). Commercial Vaccines and Vaccination Strategies Against Infectious Laryngotracheitis: What We Have Learned and Knowledge Gaps That Remain. Avian Dis..

[3] Garcia M. (2017). Current and future vaccines and vaccination strategies against infectious laryngotracheitis (ILT) respiratory disease of poultry. Vet. Microbiol..

[4] Maekawa D., Riblet S.M., Newman L., Koopman R., Barbosa T., Garcia M. (2019). Evaluation of vaccination against infectious laryngotracheitis (ILT) with recombinant herpesvirus of turkey (rHVT-LT) and chicken embryo origin (CEO) vaccines applied alone or in combination. Avian Pathol..

[5] Guy J.S., Barnes H.J., Smith L. (1991). Increased virulence of modified-live infectious laryngotracheitis vaccine virus following bird-to-bird passage. Avian Dis..

[6] Gimeno I.M., Cortes A.L., Faiz N.M., Hernandez-Ortiz B.A., Guy J.S., Hunt H.D., Silva R.F. (2015). Evaluation of the Protection Efficacy of a Serotype 1 Marek’s Disease Virus-Vectored Bivalent Vaccine Against Infectious Laryngotracheitis and Marek’s Disease. Avian Dis..

[7] Gimeno I.M., Cortes A.L., Guy J.S., Turpin E., Williams C. (2011). Replication of recombinant herpesvirus of turkey expressing genes of infectious laryngotracheitis virus in specific pathogen free and broiler chickens following in-ovo and subcutaneous vaccination. Avian Pathol..

[8] Dunn J.R., Dimitrov K.M., Miller P.J., Garcia M., Turner-Alston K., Brown A., Hartman A. (2019). Evaluation of Protective Efficacy When Combining Turkey Herpesvirus-Vector Vaccines. Avian Dis..

[9] Vagnozzi A., Zavala G., Riblet S.M., Mundt A., Garcia M. (2012). Protection induced by commercially available live-attenuated and recombinant viral vector vaccines against infectious laryngotracheitis virus in broiler chickens. Avian Pathol..

[10] Scheiermann J., Klinman D.M. (2014). Clinical evaluation of CpG oligonucleotides as adjuvants for vaccines targeting infectious diseases and cancer. Vaccine.

[11] Abdul-Cader M.S., Ahmed-Hassan H., Amarasinghe A., Nagy E., Sharif S., Abdul-Careem M.F. (2017). Toll-like receptor (TLR)21 signalling-mediated antiviral response against avian influenza virus infection correlates with macrophage recruitment and nitric oxide production. J. Gen. Virol..

[12] Farooq M., Batool M., Kim M.S., Choi S. (2021). Toll-Like Receptors as a Therapeutic Target in the Era of Immunotherapies. Front. Cell Dev. Biol..

[13] Abdul-Cader M.S., Amarasinghe A., Palomino-Tapia V., Ahmed-Hassan H., Bakhtawar K., Nagy E., Sharif S., Gomis S., Abdul-Careem M.F. (2018). In-ovo CpG DNA delivery increases innate and adaptive immune cells in respiratory, gastrointestinal and immune systems post-hatch correlating with lower infectious laryngotracheitis virus infection. PLoS ONE.

[14] Barjesteh N., Shojadoost B., Brisbin J.T., Emam M., Hodgins D.C., Nagy E., Sharif S. (2015). Reduction of avian influenza virus shedding by administration of Toll-like receptor ligands to chickens. Vaccine.

[15] Bavananthasivam J., Kulkarni R.R., Read L., Sharif S. (2018). Reduction of Marek’s Disease Virus Infection by Toll-Like Receptor Ligands in Chicken Embryo Fibroblast Cells. Viral Immunol..

[16] Bavananthasivam J., Read L., Astill J., Yitbarek A., Alkie T.N., Abdul-Careem M.F., Wootton S.K., Behboudi S., Sharif S. (2018). The effects of in-ovo administration of encapsulated Toll-like receptor 21 ligand as an adjuvant with Marek’s disease vaccine. Sci. Rep..

[17] Boone A.C., Kulkarni R.R., Cortes A.L., Gaghan C., Mohammed J., Villalobos T., Esandi J., Gimeno I.M. (2024). Evaluation of Adjuvant Effect of Cytosine-Guanosine-Oligodeoxynucleotide in Meat-Type Chickens Coadministered In-ovo with Herpesvirus of Turkey Vaccine. Viral Immunol..

[18] Gaghan C., Browning M., Cortes A.L., Gimeno I.M., Kulkarni R.R. (2023). Effect of CpG-Oligonucleotide in Enhancing Recombinant Herpes Virus of Turkey-Laryngotracheitis Vaccine-Induced Immune Responses in One-Day-Old Broiler Chickens. Vaccines.

[19] Gaghan C., Browning M., Fares A.M., Abdul-Careem M.F., Gimeno I.M., Kulkarni R.R. (2023). In-ovo Vaccination with Recombinant Herpes Virus of the Turkey-Laryngotracheitis Vaccine Adjuvanted with CpG-Oligonucleotide Provides Protection against a Viral Challenge in Broiler Chickens. Viruses.

[20] Pfaffl M.W. (2001). A new mathematical model for relative quantification in real-time RT-PCR. Nucleic Acids Res..

[21] Abdul-Cader M.S., Palomino-Tapia V., Amarasinghe A., Ahmed-Hassan H., De Silva Senapathi U., Abdul-Careem M.F. (2018). Hatchery Vaccination Against Poultry Viral Diseases: Potential Mechanisms and Limitations. Viral Immunol..

[22] Thapa S., Nagy E., Abdul-Careem M.F. (2015). In-ovo delivery of Toll-like receptor 2 ligand, lipoteichoic acid induces pro-inflammatory mediators reducing post-hatch infectious laryngotracheitis virus infection. Vet. Immunol. Immunopathol..

[23] Brownlie R., Zhu J., Allan B., Mutwiri G.K., Babiuk L.A., Potter A., Griebel P. (2009). Chicken TLR21 acts as a functional homologue to mammalian TLR9 in the recognition of CpG oligodeoxynucleotides. Mol. Immunol..

[24] Keestra A.M., de Zoete M.R., Bouwman L.I., van Putten J.P. (2010). Chicken TLR21 is an innate CpG DNA receptor distinct from mammalian TLR9. J. Immunol..

[25] Thapa S., Cader M.S., Murugananthan K., Nagy E., Sharif S., Czub M., Abdul-Careem M.F. (2015). In-ovo delivery of CpG DNA reduces avian infectious laryngotracheitis virus induced mortality and morbidity. Viruses.

[26] Borenshtain R., Witter R.L., Davidson I. (2003). Persistence of chicken herpesvirus and retroviral chimeric molecules upon in vivo passage. Avian Dis..

[27] Wolfrum N. (2020). Infectious laryngotracheitis: An update on current approaches for prevention of an old disease. J. Anim. Sci..

[28] Barjesteh N., Behboudi S., Brisbin J.T., Villanueva A.I., Nagy E., Sharif S. (2014). TLR ligands induce antiviral responses in chicken macrophages. PLoS ONE.

[29] Cicchese J.M., Evans S., Hult C., Joslyn L.R., Wessler T., Millar J.A., Marino S., Cilfone N.A., Mattila J.T., Linderman J.J. (2018). Dynamic balance of pro- and anti-inflammatory signals controls disease and limits pathology. Immunol. Rev..

[30] Baaten B.J., Li C.-R., Bradley L.M. (2010). Multifaceted Regulation of T Cells by CD44. Commun. Integr. Biol..

[31] Coppo M.J., Hartley C.A., Devlin J.M. (2013). Immune responses to infectious laryngotracheitis virus. Dev. Comp. Immunol..

[32] Maekawa D., Riblet S.M., Whang P., Hurley D.J., Garcia M. (2021). Activation of Cytotoxic Lymphocytes and Presence of Regulatory T Cells in the Trachea of Non-Vaccinated and Vaccinated Chickens as a Recall to an Infectious Laryngotracheitis Virus (ILTV) Challenge. Vaccines.

[33] Abdul-Cader M.S., De Silva Senapathi U., Nagy E., Sharif S., Abdul-Careem M.F. (2018). Antiviral response elicited against avian influenza virus infection following activation of toll-like receptor (TLR)7 signaling pathway is attributable to interleukin (IL)-1beta production. BMC Res. Notes.

[34] Krieg A.M. (2004). Antitumor applications of stimulating toll-like receptor 9 with CpG oligodeoxynucleotides. Curr. Oncol. Rep..

[35] Maekawa D., Whang P., Riblet S.M., Hurley D.J., Guy J.S., Garcia M. (2021). Assessing the infiltration of immune cells in the upper trachea mucosa after infectious laryngotracheitis virus (ILTV) vaccination and challenge. Avian Pathol..

